# Effect of Repositioning Maneuver Type and Postmaneuver Restrictions on Vertigo and Dizziness in Benign Positional Paroxysmal Vertigo

**DOI:** 10.1100/2012/162123

**Published:** 2012-09-02

**Authors:** Michel Toupet, Evelyne Ferrary, Alexis Bozorg Grayeli

**Affiliations:** ^1^Centre d'Explorations Fonctionnelles Oto-Neurologiques, Institut de Recherche en Oto-Neurologie (IRON), 75015 Paris, France; ^2^Otolaryngology Department, Beaujon Hospital, 100 Boulevard du Général Leclerc, APHP, 92118, Clichy Cedex, Inserm, UMRS-867 and Paris Diderot University, 75018 Paris, France

## Abstract

*Introduction*. To compare the efficiency of Epley (Ep) and Sémont-Toupet (ST) repositioning maneuvers and to evaluate postmaneuver restriction effect on short-term vertigo and dizziness after repositioning maneuvers by an analog visual scale (VAS) in benign positional paroxysmal vertigo (BPPV). *Material and Methods*. 226 consecutive adult patients with posterior canal BPPV were included. Patients were randomized into 2 different maneuver sequence groups (*n* = 113): 2 ST then 1 Ep or 2 Ep then 1 ST. Each group of sequence was randomized into 2 subgroups: with or without postmaneuver restrictions. Vertigo and dizziness were assessed from days 0 to 5 by VAS. *Results*. There was no difference between vertigo scores between Ep and ST groups. Dizziness scores were higher in Ep group during the first 3 days but became similar to those of ST group at days 4 and 5. ST maneuvers induced liberatory signs more frequently than Ep (58% versus 42% resp., *P* < 0.01, Fisher's test). After repositioning maneuvers, VAS scores decreased similarly in patients with and without liberatory signs. Postmaneuver restrictions did not influence VAS scores. *Conclusion*. Even if ST showed a higher rate of liberatory signs than Ep in this series, VAS scores were not influenced by these signs.

## 1. Introduction

Benign paroxysmal positional vertigo (BPPV) represents the most common etiology of vertigo and 1% of all patient visits to a physician [1, 2]. VAS has already been used to evaluate balance disorders [3, 4]. In a previous publication, we studied the validity of VAS in assessing vertigo and dizziness independently and on a daily basis after repositioning maneuvers in BBPV.

Semont-Toupet maneuver (ST) was described in 1985 [5] following Norré and De Weerdt [6] and Brandt and Daroff maneuvers in 1980 [7]. Several European centers focused on this technique [8]. Epley described his repositioning maneuver in 1992 [9], which is currently practiced by many centers worldwide [10, 11]. Centers employing both maneuvers in routine are rare as judged by the publications. Hence, the comparison of these 2 maneuvers by a referral center is rarely reported (for review see [12]).

The appearance of an intense rotatory vertigo several seconds to several minutes after the maneuver associated with an ageotropic nystagmus, also defined as liberatory vertigo, and nystagmus has been described and used as a success criterion of repositioning maneuvers [13]. A recent report showed that these signs were associated with higher recovery of posterior canal BBPV as evaluated by a negative Dix-Hallpike test 1 and 24 hours after the repositioning maneuver [14]. However, the value of this sign regarding the symptoms after the repositioning maneuver is still unclear. It appears interesting to reevaluate this criterion and to investigate the outcome of those patients who do not meet this criterion.

Postmaneuver restrictions have been proposed by the authors who described the maneuvers in order to prevent recurrence [15]. These restrictions include head movement, lying in the bed with at least 3 pillows, not lying on the side of disease, and avoiding cervical extension or rotation. Other authors criticized the efficacy of these restrictions based on the absence of proof on therapeutic efficacy and the difficulties in everyday life that they implied [10, 14–17].

The aim of this study was to compare the efficiency of ST and Ep maneuvers, to assess the value of liberatory signs in the recovery of symptoms and to evaluate the efficacy of postmaneuver restrictions by a daily VAS evaluation of vertigo and dizziness during the week following the maneuvers.

## 2. Materials and Methods

### 2.1. Population

**Figure 1:**
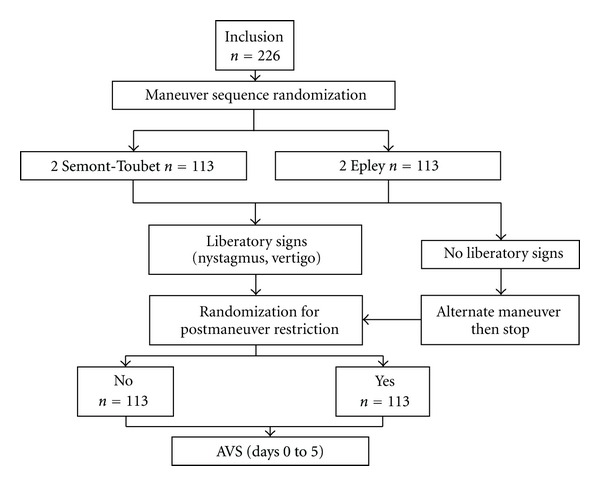
Flow chart of the study: patients were first randomized for Epley and Semont-Toupet maneuver sequences. In each group, a second randomization was performed dividing the patients into 2 subgroups: with or without postmaneuver restrictions.

Two-hundred and twenty-six consecutive adult patients suffering from a BPPV of the posterior semicircular canal on one side without any other cause of vertigo examined in one referral centre were included in this prospective study (Figure 1). Patients' informed consent was obtained and the study followed the guidelines of the institutional ethics committee. BPPV with the involvement of other canals or bilateral forms was excluded. The population comprised 171 females (76%) and 55 males (24%). The mean age was 65 years (range: 27 to 93 years). The right labyrinth was involved in 127 cases (56%) and the left in 99 cases (44%).

**Figure 2:**
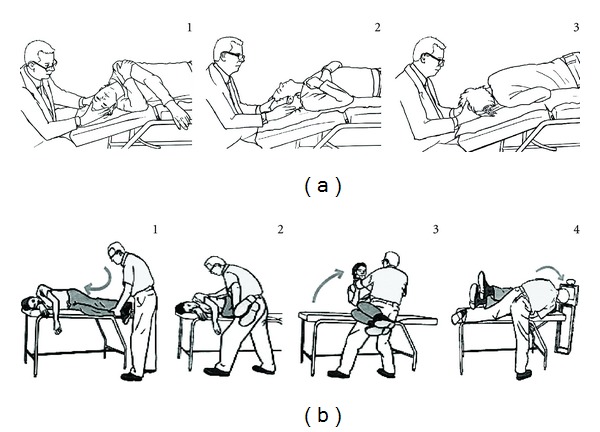
Epley (a) and Semont-Toupet (b) maneuvers for a right posterior canal BPPV. Numbers indicate the action sequence.

After a Dix-Hallpike test [18] locating the involved canal, patients were randomly assigned to or Epley (E, *n* = 113) (9, Figure 2(a)) or Semont-Toupet (ST, *n* = 113) (5, Figure 2(b)) repositioning maneuver sequences (Table 1). The presence of both liberatory nystagmus, and vertigo after the maneuver was noted. In their absence, the maneuver was repeated twice, and the interval between each maneuver was set at 7 minutes. The apparent failure was defined as the absence of liberatory nystagmus or vertigo after 2 maneuvers. In this case, the alternate maneuver was performed as a last attempt and the sequence was subsequently stopped. The diagnostic Dix-Hallpike maneuver was not repeated after the repositioning maneuvers.

### 2.2. Semont-Toupet Maneuver

The patient was positioned in lateral decubitus on the side of the disease on the examination bed, head turned upwards at 45° from the frontal plane and feet hanging on the side of the examination bed [5]. The patient held the physician's wrist by both hands and kept the elbows close to the torso. The physician held the patient's neck with both hands. The maneuver consisted of moving the patient rapidly and firmly to the opposite lateral decubitus head turned 45° downwards from the frontal plane. This movement comprised an acceleration followed by a rapid deceleration at the gentle contact between the head and the examination bed. A rotatory ageotropic (liberatory) nystagmus appeared, lasting several seconds in the majority of cases. Patient was maintained in this position during 5 minutes. The patient was then brought back to a sitting position on the side of the bed.

### 2.3. Epley Maneuver

The patient was in the supine position, head turned to the side of the disease and neck extended [9]. The physician turned the head slowly to the opposite position (in 20 seconds). Then the rotation of the shoulders and the hips to the opposite side allowed continuing the slow head rotation for 180° with a lateral decubitus on the opposite side of the disease, followed by a ventral decubitus (nose down). The patient stayed in this position for 5 minutes. A liberatory nystagmus is looked for at this time. Subsequently, the patient is brought to a lateral decubitus on the side of the disease, back to the supine position, and then to a sitting position very slowly (Figure 2).

Postmaneuver restrictions were explained to the patient and accompanied by a written memo. These instructions included sleeping with several pillows with the head in near-vertical position, avoiding head-tilts (shampoo in hair saloon, dentist), avoiding sport, and avoiding to lie down on the side of BPPV during the observation period (6 days).

As described before [19], all patients were asked to use VAS to assess their vertigo (V) and dizziness (D) separately from day 0 to day 5 following the repositioning maneuver (V0 to V5 and D0 to D5, resp.). Patients were provided with explanations to distinguish vertigo representing a “spinning sensation comparable to a merry-go-round,” from the dizziness that was defined as a “sensation of unsteadiness comparable to being placed on a moving or a rocking boat.” The VAS score sheet contained 6 pairs of columns measuring 10 centimetres printed on one page. One pair of columns represented vertigo and dizziness intensities separately for each day.

At the first visit, the scoring and the difference between vertigo and dizziness were explained to the patient. The patient rated the symptoms 15 to 30 minutes after the repositioning maneuvers in the presence of the physician (day 0). The physician explained the principle of VAS and the direction of the columns. He asked the patient to draw a horizontal bar at the level of his or her symptom on the corresponding vertical column. For the following days, the patient completed the scores at home and the document was mailed to the centre. Additionally, patients were asked to provide free written comments on their global satisfaction at the end of the observation period (day 5).

Two-hundred and three (88%) completed documents were returned to the centre. There was no difference of sex ratio, age, and proportion of liberatory signs or dizziness category at day 0 between patients who completed the VAS document and the nonrespondents (*n* = 27, data not shown).

VAS scores were measured as the distance separating the lower extremity of each column to the middle of the bar placed by the patient in millimetres in a simple blind manner without the knowledge of maneuver sequence.

### 2.4. Statistical Analysis

Clinical data and VAS scores were collected in a database. Statistical tests were carried out using Statview (SAS Institute Inc., Cary, NC). Results were expressed as mean ± SEM. *P* < 0.05 was considered as significant. Normal distribution of V and D scores at days 0 to 5 was verified (data not shown). Comparison of categorical variables in subgroups of patients was carried out by a *χ* ^2^ test. For paired comparisons between V and D categories at the same day, a paired *t*-test was used. In order to compare VAS scores (V or D) between 2 patient subgroups, an unpaired *t* (Student's) test was applied. A one-way ANOVA was chosen to compare one score in more than 2 categories of the population. For comparison between V or D scores at different days, and in more than 2 categories, a two-way ANOVA followed by a Bonferroni posttest was employed. 

## 3. Results

### 3.1. Comparison between Repositioning Maneuver Sequences

**Figure 3:**
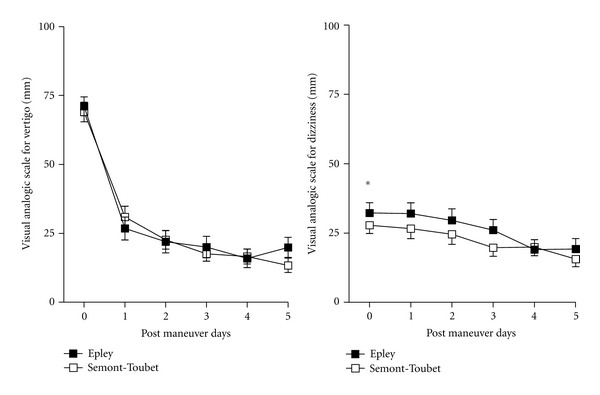
Time course of visual analog scale for vertigo and dizziness following one or two Epley or Semont-Toupet maneuvers: patients had one or two maneuvers of the same type followed by a liberatory nystagmus or vertigo (Epley, *n* = 58 or ST, *n* = 79). Patients with a third alternate maneuver were not included in this comparison. Patients with Epley maneuvers had a higher score for dizziness during the first 3 days of observation period. *P < 0.05, Epley versus ST for dizziness, 2-way ANOVA.

In case of liberatory signs after one or 2 maneuvers, VAS scores for vertigo and dizziness decreased from days 0 to 5 (Figure 3). Scores for vertigo were similar between Epley and ST groups. In contrast, dizziness scores appeared higher in Epley in comparison to ST group transiently from days 0 to 3 (Figure 3). Subsequently, dizziness scores became similar between Epley and ST groups (days 4 and 5).

### 3.2. Influence of Liberatory Nystagmus and Vertigo on VAS Scores

**Figure 4:**
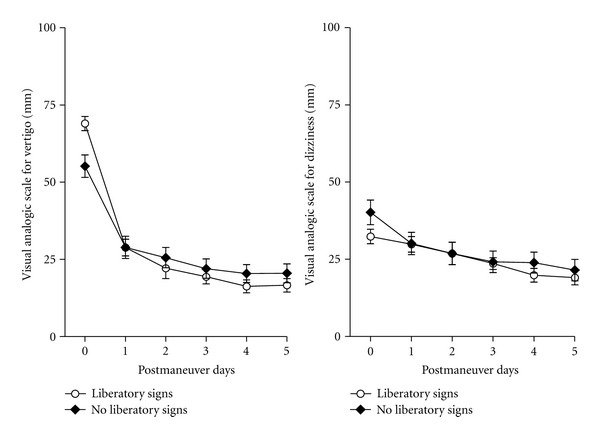
Time course of visual analog scale for vertigo and dizziness following liberatory nystagmus and vertigo: VAS was compared between groups with and without liberatory nystagmus and vertigo independently from the type of maneuver. Scores decreased in both groups with time and no difference could be observed between the 2 groups (not significant for the effect of liberatory signs and *P* < 0.0001 for the effect of time, two-way, ANOVA).

**Figure 5:**
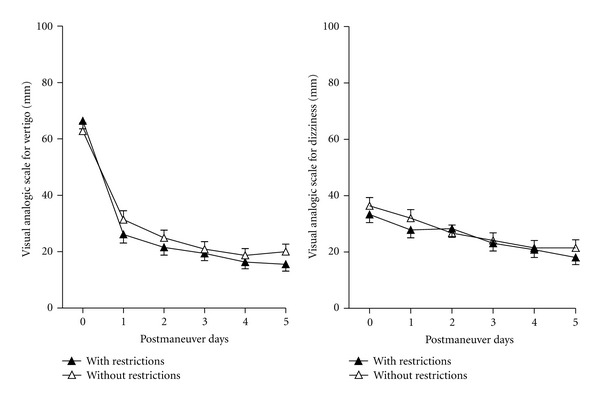
Time course of visual analog scale for vertigo and dizziness as a function of the number of therapeutic maneuvers and the presence or absence of liberatory signs at the 3rd maneuver. VAS was compared between groups with 1, 2, or 3 maneuvers with liberatory signs (vertigo and nystagmus), and 3 maneuvers without liberatory signs independently from the type of maneuver. Scores decreased in both groups with time and no difference could be observed between groups for vertigo (not significant for the effect of number of maneuvers and *P* < 0.0001 for the effect of time, two-way ANOVA). Patients with 2 or 3 maneuvers had higher dizziness scores than those with only one maneuver (*P < 0.01 for the effect of number of maneuvers and *P* < 0.0001 for the effect of time, two-way ANOVA).

The proportion of cases with liberatory nystagmus and vertigo was similar between the two groups (Table 2). Liberatory nystagmus and vertigo were more frequently observed after ST than after Epley after two same maneuvers (70% versus 51%, *P* < 0.001, Fisher's exact test). ST as a 3rd alternate maneuver yielded a higher rate of liberatory signs than Epley (12%, versus 3%, *P* < 0.02, Fisher's exact test). However, VAS for vertigo and dizziness did not seem to be influenced by the liberatory signs (Figure 4). The number of therapeutic maneuvers and the presence or the absence of liberatory signs after the 3rd maneuver did not influence the vertigo scores (Figure 5, not significant for number of maneuvers, and *P* < 0.001 for the effect of time, 2-way ANOVA). In contrast, dizziness scores seemed to be influenced by the number of therapeutic maneuvers, and patients with 2 or 3 maneuvers scored their dizziness higher than those who had only one (Figure 5, *P* < 0.01 for the effect of maneuver number, and *P* < 0.0001 for the effect of time, 2-way ANOVA). This observation suggests that the number of maneuvers does not influence the perceived vertigo intensity and unfavorably affects the perceived dizziness during the 5 days following the maneuvers.

### 3.3. Effect of Postmaneuver Restrictions on Vertigo and Dizziness VAS Scores

**Figure 6:**
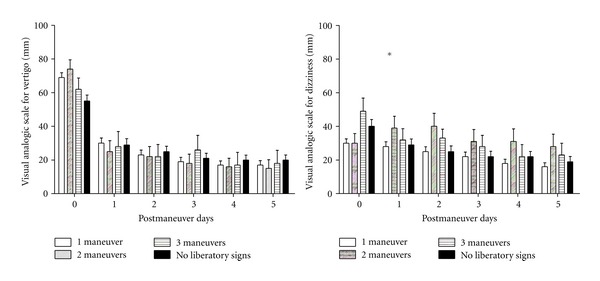
Time course of visual analog scale with or without postmaneuver restrictions independently from the maneuver type and liberatory nystagmus. No difference could be observed in the evolution of the symptoms between the two groups (not significant, two-way ANOVA).

Postmaneuver restrictions did not seem to influence VAS scores for vertigo and dizziness during the 6 postmaneuver days (Figure 6). Analyzing VAS scores separately in Ep and ST groups did not show an effect of postmaneuver restriction (data not shown).

Moreover, no effect of restriction could be evidenced by analyzing patients with or without liberatory signs separately (data not shown).

## 4. Discussion

The efficacy of repositioning maneuvers in VPPB is now well established. The manoeuvres are significantly more effective than sham but additional exercise by the patient repeating Epley maneuvers at home does not add to treatment effectiveness [11]. Liberatory nystagmus and vertigo have been generally accepted as indicators of successful otolith repositioning [13]. The notion that liberatory nystagmus and vertigo sign the efficacy of the repositioning maneuvers was first advanced by Toupet and Semont [5], Semont et al. [20], and Epley [9]. However, to our knowledge, the predictive value of these events on the postmaneuver balance disorders has not been studied. In our series, we observed that ST maneuvers led more frequently to liberatory vertigo and nystagmus than Ep but that these signs did not influence the VAS scores of vertigo or dizziness during the following days. A higher frequency of liberatory nystagmus and vertigo during ST maneuvers could simply be related to a more rapid displacement of otoconia, considering that ST maneuver provides a higher acceleration to the semicircular canals.

In this study, we performed several maneuvers during the same session. This procedure was based on the observations that repeated Epley manoeuvres in fewer sessions render more positional nystagmus-free patients when compared to those submitted to more sessions of single maneuvers [21]. However, we showed that multiple maneuvers did not enhance efficacy as measured by VAS scores and even increased the perceived dizziness during the 5 days following the therapeutic maneuver. This observation indicates that only one maneuver can be administered systematically and a second maneuver can be decided several days after depending on the symptoms.

We showed that the efficacy of Epley was similar to ST maneuver in terms of VAS of vertigo and dizziness at the end of the observation period. However, patients treated with one or two Epley maneuvers had higher scores of dizziness than patients undergoing ST during the 3 postmaneuver days. The pathophysiology of postmaneuver dizziness is not clearly understood [22]. It can be hypothesized that the return of displaced otoliths on the utricular macula leads to a relatively prolonged disturbance of utricular activity. The symptoms might vary depending on the quantity and the location of otoconia deposit. Another possible explanation can be that the canal function recovery induces a regressive dizziness. Anyhow, a central adaptation appears to progressively reduce the dizziness during the week following the maneuver. The difference of dizziness scores between ST and Ep groups might be related to differences in the dynamics of otoconia displacement and has to be further investigated.

Various postmaneuver restrictions (e.g., sleeping with several pillows with the head in near-vertical position, avoiding head-tilts and sport) are routinely prescribed after a repositioning maneuver. Studies on their efficacy are contradictory [10, 14–17]. Several studies had methodological limitations: not randomized [16] or vertigo only evaluated by interrogation [14, 15, 17]. Objective outcome measures were Dix-Hallpike test [14] and the number of maneuvers necessary to cure the symptoms [15, 16]. In our study, we investigated the effect of a prolonged restriction (7 days) in a randomized manner in both Ep and ST groups, and we did not observe any effect of restriction on vertigo and dizziness scores. This result is in accordance with a recent report in an experimental model in frogs showing that otoconies are stably replaced 3 to 5 minutes after a repositioning maneuver [23].

In conclusion, ST and Ep maneuvers had a similar efficacy in reducing VPPB vertigo and dizziness. Repetition of maneuvers did not influence vertigo scores but appeared to increase dizziness during the following days. Liberatory vertigo and nystagmus did not seem to influence the outcome in terms of vertigo and dizziness after the maneuvers. Postmaneuver restrictions did not modify the intensity of vertigo and dizziness during the observation period of one week after the repositioning maneuver.

## Figures and Tables

**Table 1:** Characteristics of the 2 groups treated by different repositioning maneuver sequences: Patients were treated either by 2 Epley (Ep) maneuvers then 1 Semont-Toupet (ST), or 2 ST then 1 Ep. Each patient had a maximum of 3 maneuvers. The sequence was interrupted when a liberatory nystagmus and/or vertigo were observed. In the absence of these two signs, a maximum of 3 maneuvers were performed. There was no difference between group characteristics.

	2 Ep-1 ST (*n* = 113)	2ST-1Ep (*n* = 113)
Sex ratio (M/F)	0.38	0.27 (ns)
Age	66 ± 1.3	63 ± 1.3 (ns)
BBPV side		
Right	67	60
Left	46	53
Vertigo intensity at day 0 (VAS, mm)	65.8 ± 2.65	63.4 ± 3.04 (ns)

ns: Not significant, unpaired *t*-test.

**Table 2:** Comparison of liberatory signs in Epley (Ep) and Semont-Toupet (ST) maneuver sequences. The sequence was stopped after vertigo and nystagmus. These signs more frequently observed after ST than after Ep (70% versus 51%, *P* < 0.001, Fisher's exact test). ST as a 3rd maneuver also yielded a higher rate of liberatory signs than Ep (12%, versus 3%, *P* < 0.02, Fisher's exact test).

	Maneuver sequences	
	Epley (*n* = 113)	Semont-Toupet (*n* = 113)
Liberatory signs after 1st maneuver	Ep: 45 (40%)	ST: 65 (58%)
Liberatory signs after 2nd maneuver	Ep: 13 (12%)	ST: 14 (12%)^∗∗∗^
Liberatory signs after alternate maneuver	ST: 14 (12%)^∗^	Ep: 3 (3%)
No liberatory signs after alternate maneuver	41 (36%)	31 (27%)

^∗∗∗^ *P* < 0.001, proportion of liberatory signs after one or two ST versus Ep maneuvers, Fisher's exact test.

^∗^ *P* < 0.05, proportion of liberatory signs after alternate ST maneuver versus Ep, Fisher's exact test.
